# Stress and Anxiety Levels in Pregnant and Post-Partum Women during the COVID-19 Pandemic

**DOI:** 10.3390/ijerph17249450

**Published:** 2020-12-17

**Authors:** Anna Stepowicz, Barbara Wencka, Jan Bieńkiewicz, Wojciech Horzelski, Mariusz Grzesiak

**Affiliations:** 1 Department of Perinatology, Obstetrics and Gynecology, Polish Mother’s Memorial Hospital—Research Institute, 93-338 Lodz, Poland; bwencka@gazeta.pl (B.W.); mariusz.grzesiak@gmail.com (M.G.); 2 Department of Operative Gynecology, Endoscopy and Gynecologic Oncology, Polish Mother’s Memorial Hospital—Research Institute, 93-338 Lodz, Poland; jan.bienkiewicz@iczmp.edu.pl; 3 Faculty of Mathematics and Computer Science, University of Lodz, 90-136 Lodz, Poland; wojciech.horzelski@wmii.uni.lodz.pl; 4 Department of Gynecology and Obstetrics, Medical University, 90-419 Lodz, Poland

**Keywords:** distress, stress, anxiety, pregnancy, post-partum, COVID-19, mental health

## Abstract

The aim of this study was to analyze stress and anxiety levels experienced by pregnant and post-partum women during the COVID-19 pandemic, as well as to indicate the social and medical factors that could contribute to stress and anxiety. A total of 210 patients were enrolled in the study. Two well-established test-tools were applied: State-Trait Anxiety Inventory (STAI) and Perceived Stress Scale (PSS-10). The study revealed that the levels of stress and anxiety experienced by the surveyed patients were moderate to high. We demonstrated that women with mental treatment history, those in the first trimester of pregnancy and the ones that are single or in an informal relationship tend to experience higher levels of psychological distress and anxiety. Such factors as age, education, parity, eventful obstetric history, comorbidities, and the number of hospital stays proved to be statistically insignificant in the analysis. Our findings could be used to identify patients at greater risk of experiencing adverse mental effects and to provide them with adequate psychological support. Further multi-center studies are warranted in order to draw final conclusions.

## 1. Introduction

The COVID-19 pandemic is a new and rapidly evolving health problem which currently affects the lives of most people worldwide. The novel coronavirus outbreak, which started in Wuhan, China in December 2019, and then spread all over the world during the spring of 2020, is now hitting its second wave, with skyrocketing daily cases in most European countries and spiking death rates that follow. Apart from being a serious health issue to the infected individuals themselves, it is a huge burden on health systems and economies [1]. Not only does COVID-19 alter our everyday routine and behavior, but it undoubtedly presents a great psychological strain [2], which could also result in PTSD development in vulnerable individuals [3].

Pregnancy is a rewarding yet challenging period of life, which demands physical, psychological and social adjustment to a new role. In general, pregnant women are not prone to experiencing higher levels of stress and anxiety in comparison to non-pregnant controls [4,5]. Nevertheless, women with complicated pregnancies report higher levels of anxiety symptoms compared to low-risk pregnant subjects [6,7]. Literature data on the impact of COVID-19 infection on pregnancy outcome and its long-time influence on the neonate and infant are still rather scarce. However, it has been said that it significantly increases the risk of preterm birth (approximately three-fold), mostly due to iatrogenic reasons. It was also shown that COVID-19 during pregnancy is associated with higher rates of caesarean sections [8,9]. Generally, psychological distress is believed to play an important role in the occurrence of adverse pregnancy outcomes, preterm delivery included [10,11,12]. Although advanced age and comorbidities are the greatest risk factors of developing serious medical conditions and even death due to coronavirus-induced bilateral pneumonia [13], some physiological changes in pregnancy also increase susceptibility to infections and encourage rapid progression to respiratory failure. Moreover, T-helper 2 (Th2) system dominance, which is typical in pregnancy, leaves the mother-to-be vulnerable to viral infections in general [14]. On the other hand, observational studies show that the clinical appearance of SARS-CoV-2 infection and the likelihood of contracting it is similar in pregnant and non-pregnant women age- and health-status matched [15]. Literature data also indicate that most pregnant individuals with COVID-19 present no characteristic symptoms at all, and such manifestations as fever, myalgia or cough are less likely to occur than in non-pregnant controls. When symptoms in pregnancy do occur, they mostly resemble mild to moderate flu [8]. Interestingly, a paper from France suggested that contracting COVID-19 infection in the second half of pregnancy increases the risk of admission to ICU- by 5 times in comparison to patients less than 20-weeks pregnant [16]. Although the data concerning the psychological effect of the coronavirus pandemic on pregnant women are limited, most studies suggest that it has a moderate to even severe impact [17,18,19,20]. Pregnant women tend to worry in general and perceive their situation during the pandemic in a negative manner, often due to the lack of consistent and validated information about the impact on pregnancy itself. Interestingly, it was also shown that women in the first trimester are more prone to experiencing high levels of anxiety and emotional distress in comparison to their counterparts in more advanced stages of pregnancy [18]. Certain studies suggest that higher education could be a risk factor of high anxiety levels in pregnant individuals [17]. Notably, there are reports which suggested that despite anxiety levels during the pandemic being higher in general population, they are still lower in pregnant women than in their non-pregnant counterparts [21]. Moderate physical activity during pregnancy is believed to play an important role in decreasing stress, depression, and anxiety levels [22].

In order to assess stress and anxiety levels in pregnant women, various non-invasive standardized psychological assessments can be employed: State-Trait Anxiety Inventory (STAI), 1–10 Visual Analogue Scale (VAS, referred to as the Stress Scale), Perceived Stress Scale (PSS-10), Beck Anxiety Inventory, the anxiety subscale of the Hospital Anxiety and Depression Scale, etc. [23]. Moreover, it is also possible to analyze serum and/or saliva levels of stress hormones, such as cortisol, CRH, catecholamines (adrenaline, noradrenaline), growth hormone and prolactin.

## 2. Aim of the Study

The purpose of the study was to analyze the degree of stress and anxiety experienced by pregnant and post-partum women during the COVID-19 pandemic. We also aimed to identify both social factors and medical conditions that could contribute to stress and anxiety levels in these patients. The following aspects were investigated in terms of their correlation with anxiety and stress levels: age, education, marital status, parity, eventful obstetric history, the history of psychiatric treatment, number of hospital stays during current pregnancy, gestational age/post-partum, the presence of comorbidities, and the time of assessment.

## 3. Materials and Methods

The study was conducted in the Department of Perinatology, Obstetrics and Gynecology, Polish Mother’s Memorial Hospital and Research Institute, Lodz, Poland between 7 April and 24 May 2020. Two hundred and ten adult females aged 19 to 45, both pregnant and post-partum subjects, hospitalized in our department during the abovementioned period, were enrolled.

To begin with, participants were asked to fill in a short 9-item questionnaire on basic social and medical factors: age, education, marital status, parity, presence of eventful obstetric history, history of mental treatment, number of hospital stays during the current pregnancy, gestational age (or post-partum), and presence of comorbidities.

Then, in order to credibly and quantitatively assess stress and anxiety levels, we applied two well-established test-tools: State-Trait Anxiety Inventory (STAI) and Perceived Stress Scale (PSS-10). They are non-invasive, easy and fast to conduct (5–10 min) self-report questionnaires, which are also supported by strong psychometric data. Both tools were applied in the Polish validated version.

The State-Trait Anxiety Inventory allows measurement via self-report of the presence and severity of current symptoms of anxiety and a general propensity to be anxious. There are 2 subscales within this test, both consisting of 20 separate items on a 4-point scale. The State Anxiety Scale (X-1) evaluates the current state of anxiety using items that measure subjective feelings of apprehension, tension, nervousness, worry, and activation/arousal of the autonomic nervous system. On the other hand, the Trait Anxiety Scale (X-2) evaluates relatively stable aspects of “anxiety proneness,” including general states of calmness, confidence, and security [23]. The PSS-10 assesses the degree to which one appraises their life situation as stressful, unpredictable, uncontrollable, and overloading over the previous month via ten items on a 5-point Likert scale [24]. For both scales, some items are reverse-coded and responses are summed for total scores with higher scores indicating greater intensity of symptoms. The results of these tests are presented both in point- and STEN-scores. STEN score (standard ten score) is a standardized 10-point instrument applied in psychometric tests with a mid-point for population of 5.5 (between STEN 5 and 6) and a standard deviation of 2. We used standard score cut-offs on each instrument to denote low/moderate/high stress symptoms.

The design of the study was accepted by the Local Ethical Committee of Polish Mother’s Memorial Hospital and Research Institute in full compliance with the Institute’s policy concerning questionnaire surveys (10 April 2020). All patients received detailed information about the design and purpose of the study and gave their written consent to participate. They were also informed about the possibility to withdraw their consent at any time.

The collected data were analyzed statistically using frequency distribution table and chi-squared test. A 2-sided *p* value less than 0.05 was considered statistically significant.

## 4. Results

The median age of patients was 31 years (19 to 45 years old). In the study group, 164 women were pregnant: 11 in the first trimester (5.2%), 46 in the second trimester (21.9%) and 107 in the third trimester (51.0%), whereas 46 were post-partum (21.9%). The majority of patients enrolled in the study were nulliparous: 112 (46%). There were 66 women (31.4%) in their second pregnancy, 24 (11.4%) in their third pregnancy, 6 in their fourth pregnancy and 2 in their fifth pregnancy. For 50% of patients (105), it was their first stay at the hospital during pregnancy, 51 individuals (24.4%) reported it as their second stay, 39 (18.7%) declared it as their third stay, while the rest (6.7%) were hospitalized four or more times. The presence of pregnancy-associated comorbidities was reported by 113 out of 210 patients (53.8%). Complicated obstetric history was defined in the protocol as the number of past miscarriages (pregnancy losses up to 22 weeks of gestation): the majority of 163 patients (77.6%) had no history of previous miscarriage, 32 (15.2%) experienced one miscarriage, 10 (4.85%) had two such events and 5 (2.4%) had three or more. The history of previous or ongoing mental treatment, defined as both psychotherapy and/or use of pharmaceuticals, was reported by 29 patients (13.8%). Regarding the level of education, the distribution was as follows: 15 (7.1%) declared basic education, 72 (34.3%) secondary education, while 123 (58.6%) declared higher education. As for marital status, 147 patients (70%) were married, 62 (29.5%) were single or in an informal relationship, and only 1 (0.5%) was divorced. One hundred and ten subjects (52.4%) were questioned in April 2020, whereas 100 (47.6%) filled in the self-report questionnaires in May.

The median score of PSS-10 test among patients was 18 points in a 0–40-point scale (SD +/− 6.59) and is perceived as a moderate level of stress experienced in the past month. The median score of the STAI-trait subscale was 43 points (SD +/− 8.59), suggesting moderate anxiety proneness among the study group. The mean score of STAI-state subscale, however, was as high as 45 points (SD +/− 11.68) and indicated high levels of anxiety defined as current feeling of tension, nervousness, and general worry.

Table 1 presents the summary of the abovementioned findings.

The results of our study reveal a statistically significant correlation between the history of mental treatment and high levels of anxiety in STAI-trait (*p* = 0.0001): 69% of patients with such history showed high levels of anxiety in comparison to only 29% in the subgroup with no such treatment (Table 2). Similar results were acquired based on PSS-10 (*p* = 0.0062): high levels of stress were much more common in patients with mental treatment history (69%) than in those without one (39%) (Table 3). This correlation, however, was not shown in STAI-state. Moreover, we found a statistically significant correlation between marital status and anxiety levels measured by STAI-state (*p* = 0.02), which showed that anxiety levels in pregnancy are higher in single women and in those in informal relationship compared to married controls. High levels of anxiety were found in 55% of married pregnant women, compared to as many as 71% of their counterparts who were single/in an informal relationship (Table 4). We did not find marital status to be a statistically significant stress and anxiety factor based on PSS-10 or STAI-trait, though. Finally, we demonstrated a statistically significant correlation between gestational age and anxiety levels based on STAI-state (*p* = 0.0299), but not on STAI-trait or PSS-10. In patients in the first trimester, 82% demonstrated high anxiety levels, compared to 74% in the second trimester, and to 54% in the third trimester, and to only 52% in puerperium (Table 5). According to the results of our study, such factors as age, education, parity, eventful obstetric history, comorbidities, and the number of hospital stays occurred to be statistically insignificant. Table 2, Table 3, Table 4 and Table 5 depict and summarize our statistically significant findings.

## 5. Discussion

In our cross-sectional survey study, we aimed to analyze the effects of the pandemic on the psychological rather than physical condition of pregnant and postpartum women. We assumed that despite the likelihood that the majority of population will avoid serious SARS-CoV-2-induced medical complications, there is a high likelihood people will experience indirect disturbing effects of the pandemic, i.e., various restrictions (quarantine, isolation, lockdown) and emotional burden. Some studies on the general population have already confirmed the moderate negative impact of the pandemic on mental health [25]. Bearing in mind the WHO definition: ‘Health is a state of complete physical, mental and social well-being and not merely the absence of disease or infirmity’ and assuming that the aforementioned may exert a powerful negative effect on psychological health, we have found it worth the effort to perform a scientific analysis in that field. Data were collected during April and May 2020 when there was still scarce information on the virus itself, the potential scale of its outbreak in Europe and possible cures, which all added up to the body of general social distress and insecurity.

In our study we have shown that anxiety levels in pregnant and post-partum women during the COVID-19 pandemic are significantly higher (STEN 7, mean: 45 points) by means of STAI-state subscale (see Table 1). Saccone, in his research conducted on a group of 100 pregnant women in Italy, obtained very similar results to ours with a significantly elevated mean STAI score of 45 points [18]. Our findings are supported by the conclusions of Mappa’s study [17], who even suggested a double-fold increase in the number of pregnant women who experience abnormal levels of stress and anxiety during the pandemic. Moreover, the Turkish authors who examined the same population of pregnant women prior and during the COVID-19 pandemic concluded that depression and anxiety levels were significantly increased in the latter period [19]. Although the study group was rather small (63) and different test-tools than ours were applied (Inventory of Depression and Anxiety Symptoms II (IDAS II) and Beck Anxiety Inventory (BAI)), the study clearly indicated the role of this pandemic in aggravation of stress and anxiety levels.

Furthermore, both in our study and the one by Saccone [18], it was shown that anxiety levels experienced by women in the first trimester are higher than in later stages of pregnancy or post-partum. This finding is quite surprising, as one could expect the peripartum period is the most stressful and filled with uncertainty, especially when, due to various restrictions, direct contact with family members during labor was banned. However, in our study, the number of patients in the first trimester was rather scarce (5.2%), which is an obvious limitation of the analysis.

Moreover, it came to us as no surprise that anxiety and emotional distress were higher in patients with mental treatment history than in those without such. The correlation was statistically significant in PSS-10 and STAI-trait scale. STAI-trait shows a general propensity to experience anxiety, rather than current feeling of arousal and distress, which is better demonstrated in STAI-state scale. According to some studies, STAI did not differentiate anxious from depressed patients in a credible manner [26], which is why our result suggesting that patients with mental treatment history show higher levels of anxiety could be subject to bias. On the other hand, PSS-10, which assesses the level of psychological distress and the perception of current situation as overloading and uncontrollable, also shows that patients with positive history of mental treatment are more prone to feel stressed and overwhelmed. Our findings are consistent with another study, which also showed that previous psychiatric diagnosis, as well as low income could play an important role in the aggravation of anxiety symptoms [27].

Uncertainty and instability that may accompany a single mother are clear and obvious aspects that trigger anxiety and distress. On the contrary, a stable and happy relationship is likely to wake a feeling of calmness, security and predictability. We showed that married women tend to experience less anxiety than those who are single or in an informal relationship. We did not, however, differentiate between single pregnant patients and those in a non-legalized relationship. It could be worthwhile to analyze whether individuals in a legalized and not-legalized relationships differ in the levels of experienced stress and anxiety.

In our study, we did not find any statistically significant correlation between age of pregnant and post-partum women and anxiety levels. On the other hand, some articles show that older maternal age could be perceived as a protective factor against stress and anxiety [7,28]. The available research data, mainly on the general population, seem to be incoherent as to the role of age in experiencing high levels of stress and anxiety during the pandemic. Some survey-studies suggested higher levels of anxiety were found in younger responders [29], whereas others reported the highest anxiety levels in elderly people [25].

Although our results suggest there is no correlation between education and stress levels during the COVID-19 pandemic, there are studies on pregnant subjects that indicated lower levels of education tend to trigger higher anxiety and stress levels [30]. On the contrary, others suggested that higher education is a risk factor and increases the propensity to experience anxiety [17].

What is more, our study was conducted in the Department of Perinatology and Gynecology, Polish Mother’s Memorial Hospital and Research Institute, which is a tertiary care unit. Patients that are hospitalized in our Department are mostly in complicated pregnancies and therefore could be more prone to experience higher levels of anxiety and distress due to pregnancy-related health issues. For a better standardization of results, a large multicenter study is warranted.

To sum up, in order to draw final conclusions about the impact of SARS-COV-2 epidemic on the mental health of pregnant women, we believe that it would be of great scientific value to conduct our study again after the COVID-19 pandemic is over and to compare the results.

## 6. Conclusions

The levels of stress and anxiety in pregnant and post-partum women during the COVID-19 pandemic are moderate to high. Anxiety and distress tend to be higher in women with psychiatric treatment history, those in the first trimester of pregnancy, and the ones that are single or in an informal relationship. Our findings could be applied to identify patients at greater risk of experiencing adverse mental effects and enable us to provide them with adequate psychological support. The power of these conclusions is limited due to the single-center study design and the lack of a control group outside the period of the COVID-19 pandemic.

## Figures and Tables

**Table 1:** Results’ summary.

*n* = 210	Mean	Median	SD ^2^	SEM ^3^
PSS points	18.47	18	6.59	0.45
PSS—STEN ^1^	6	6	1.93	0.13
STAI—trait—points	43.7	43	8.59	0.59
STAI—trait—STEN ^1^	5	6	2.14	0.15
STAI—state—points	45.19	43	11.68	0.81
STAI—state—STEN ^1^	7	7	2.22	0.15

^1^ Standard Ten; ^2^ Standard Deviation; ^3^ Standard Error of the Mean.

**Table 2:** The influence of mental treatment history on anxiety levels using STAI-TRAIT subscale. *p* = 0.0001.

Stai-Trait		Mental Treatment	
Anxiety Level		NO	YES
LOW	64 (30.5%)	62	2
MEDIUM	73 (34.8%)	66	7
HIGH	73 (34.8%)	53	20
		(29%)	(68.9%)
Total	210	181	29

**Table 3:** The influence of mental treatment history on stress levels using PSS-10. *p* = 0.0062.

PSS-10		Mental Treatment	
Stress Level		NO	YES
LOW	53 (25.2%)	51	2
MEDIUM	66 (31.4%)	59	7
HIGH	91 (43.3%)	71	20
		(39.2%)	(68.9%)
Total	210	181	29

**Table 4:** The correlation between marital status and anxiety levels based on STAI-STATE subscale. *p* = 0.0222.

Stai-State		Marital Status		
Anxiety Level		Married	Single/Informal Relationship	Divorced
LOW	30 (14.3%)	21	8	1
MEDIUM	55 (26.2%)	45	10	0
HIGH	125 (59.5%)	81	44	0
		(55.1%)	(70.9%)	
Total	210	147	62	1

**Table 5:** The correlation between gestational age and anxiety levels based on STAI-STATE subscale. *p* = 0.0299.

Stai-State		Gestational Age			
Anxiety Level		1st Trimester	2nd Trimester	3rd Trimester	Puerperium
LOW	30 (14.3%)	0	1	18	11
MEDIUM	55 (26.2%)	2	11	31	11
HIGH	125 (59.5%)	9	34	58	24
		(81.8%)	(73.9%)	(54.2%)	(52.2%)
Total	210	11	46	107	46
